# Three Different Types of β-Glucans Enhance Cognition: The Role of the Gut-Brain Axis

**DOI:** 10.3389/fnut.2022.848930

**Published:** 2022-03-03

**Authors:** Minmin Hu, Peng Zhang, Ruiqi Wang, Menglu Zhou, Ning Pang, Xiaoying Cui, Xing Ge, Xiaomei Liu, Xu-Feng Huang, Yinghua Yu

**Affiliations:** ^1^Jiangsu Key Laboratory of Immunity and Metabolism, Department of Pathogen Biology and Immunology, Xuzhou Medical University, Xuzhou, China; ^2^Tianjin Third Central Hospital, Tianjin, China; ^3^Queensland Centre for Mental Health Research, Wacol, QLD, Australia; ^4^Queensland Brain Institute, The University of Queensland, St Lucia, QLD, Australia; ^5^Illawarra Health and Medical Research Institute (IHMRI) and School of Medicine, University of Wollongong, Wollongong, NSW, Australia

**Keywords:** β-glucans, cognition, microglia, gut-brain axis, oat, mushroom, curdlan

## Abstract

**Background:**

Dietary fiber is fermented in the lower gastrointestinal tract, potentially impacting the microbial ecosystem and thus may improve elements of cognition and brain function via the gut-brain axis. β-glucans, soluble dietary fiber, have different macrostructures and may exhibit different effects on the gut-brain axis. This study aimed to compare the effects of β-glucans from mushroom, curdlan and oats bran, representing β-(1,3)/(1,6)-glucan, β-(1,3)-glucan or β-(1,3)/(1,4)-glucan, on cognition and the gut-brain axis.

**Methods:**

C57BL/6J mice were fed with either control diet or diets supplemented with β-glucans from mushroom, curdlan and oats bran for 15 weeks. The cognitive functions were evaluated by using the temporal order memory and Y-maze tests. The parameters of the gut-brain axis were examined, including the synaptic proteins and ultrastructure and microglia status in the hippocampus and prefrontal cortex (PFC), as well as colonic immune response and mucus thickness and gut microbiota profiles.

**Results:**

All three supplementations with β-glucans enhanced the temporal order recognition memory. Brain-derived neurotrophic factor (BDNF) and the post-synaptic protein 95 (PSD95) increased in the PFC. Furthermore, mushroom β-glucan significantly increased the post-synaptic thickness of synaptic ultrastructure in the PFC whilst the other two β-glucans had no significant effect. Three β-glucan supplementations decreased the microglia number in the PFC and hippocampus, and affected complement C3 and cytokines expression differentially. In the colon, every β-glucan supplementation increased the number of CD206 positive cells and promoted the expression of IL-10 and reduced IL-6 and TNF-α expression. The correlation analysis highlights that degree of cognitive behavior improved by β-glucan supplementations was significantly associated with microglia status in the hippocampus and PFC and the number of colonic M2 macrophages. In addition, only β-glucan from oat bran altered gut microbiota and enhanced intestinal mucus.

**Conclusions:**

We firstly demonstrated long-term supplementation of β-glucans enhanced recognition memory. Comparing the effects of β-glucans on the gut-brain axis, we found that β-glucans with different molecular structures exhibit differentia actions on synapses, inflammation in the brain and gut, and gut microbiota. This study may shed light on how to select appropriate β-glucans as supplementation for the prevention of cognitive deficit or improving immune function clinically.

## Background

There is increasing evidence that the consumption of dietary fiber affects gut microbiota composition, and consequently impacts human health, including brain function. Previous clinical studies found that the consumption of dietary fiber is positively correlated with cognition in mid-age or elderly people (1, 2). Dietary fiber β-glucan is a long-chain polysaccharide that connects D-glucose monomers through β-glycosidic linkages. β-glucan is present in the cell walls of many natural sources, including edible mushrooms such as shiitake, reishi and ganoderma applanatum; cereal grains such as oats, barley, wheat, and rye; and bacteria and yeast such as *Saccharomyces cerevisiae, Agrobacterium, Aspergillus and Agaricus* species and algae (3, 4). The structures of β-glucans vary according to the different sources. β-glucans from mushroom and yeast consists of β-(1,3)/(1,6)-glucans that are short β (1,6)-linked branches from a β (1,3) backbone, while β-glucans from cereal (oat and barley) are β-(1,3)/(1,4)-glucans, linear β (1,4) linkages separating shorter chain of β (1,3) structures (5). Curdlan [isolated from the cell wall of the bacterium *Agrobacterium* and *Alcaligenes faecalis* (6, 7)] or β-glucans from other bacteria are linear β-(1,3)-glucans. A plethora of research works has shown that linkage type, linkage ratio and length of β-glucans impact their solubility, viscosity and aggradation and thus influence their functionalities. It is hypothesized that different types of β-glucans might have differential effects on the gut microbiome and brain functions, but this has not been thoroughly investigated.

Accumulating evidence has shown that the gut microbiota plays a crucial role in several neurological disorders. The microbiota communicates with the brain via various routes, including the immune system, the vagus and enteric nervous system and microbial-derived metabolites such as short-chain fatty acids (SCFAs) (8, 9). Besides, signaling within the gut-brain axis is also modulated by the intestinal barrier, in which the permeability is influenced by gut microbiota or gut microbiota metabolites (9). The composition and abundance of the gut microbiota are regulated by the diets, and recent research tempts to understand the impact of different diets on the gut microbiota, intestinal permeability and brain function.

The effects of β-glucans on the gut microbiota and immune response had been investigated, but many of these studies only focus on one type of β-glucans. For instance, Mitsou's study has observed an increase of Bifidobacteria and Bacteroides in aging human feces after consumption of barely β-glucan (10). Supplementation of barley β-glucan results in enrichment in lactobacilli in the rat cecum as these bacteria utilize oligosaccharides in β-glucan hydrolysates (11). However, how β-(1,3)/(1,4)-glucan from cereals affects intestinal immune response is rare reported. On the other side, a few studies show that the β-(1,3)/(1,6)-glucan from mushroom or β-(1,3)-glucan from fungi or bacteria bind to Dectin-1 receptor and is captured by gut-associated lymphoid tissue-associated immune cells and/or epithelial cells (5, 12, 13). β-(1,3)/(1,6)-glucan attenuates lipopolysaccharide (LPS)-induced activation of pro-inflammatory TLR4/MyD88/NF-κB signaling pathway and promotion of pro-inflammatory cytokines TNF-α and IL-1β expression (14, 15). However, the effects of β-(1,3)/(1,6)-glucan or β-(1,3)-glucan on the gut microbiome remain elusive.

Mushroom and cereal are popular healthy food (5, 16). Accumulating research has provided evidence for the protective effects of mushrooms and cereal against various chronic diseases and neurodegeneration disorders. A community-based cross-sectional study in Singapore demonstrates that participants who consumed mushrooms >2 portions per week have a reduced risk of mild cognitive impairment (17). Breakfast cereal consumption by children and adolescence is associated with greater cognitive performance (18, 19). A randomized, double-blind, placebo-controlled study found that chronic supplementation of green oat extract results in dose-dependent improvements in cognitive function in healthy humans (20). These findings indicate that the bioactive compounds from mushroom and oat potentially delay neurodegeneration. Furthermore, β-glucans from mushrooms, oat and curdlan are widely used in the medicine and food industry (21–23). For example, β-glucans from Shiitake mushrooms are manufactured as adjunctive medicine to treat cancers in China and Japan (21). β-glucans from whole oats, oat bran, and whole oat flour have been approved to be a health claim that reduces the risk of coronary heart disease by the US Food and Drug Administration (22). Curdlan, β-(1,3)-glucan, has heat-induced gelling properties and is widely used as a food additive; however, its effects on the gut and brain are rarely reported.

Collectively, three different types of β-glucans have shown benefits on brain function, gut microbiome and immune response. The aim of the present study was to systematically investigate whether three types β-glucans, β-(1,3)/(1,4)-glucan (oats bran), β-(1,3)/(1,6)-glucan (mushroom) and β-(1,3)-glucan (curdlan) differentially affect cognitive function, synaptic ultrastructure and microglia status as well as gut microbiota profiles and intestinal immune response.

## Materials and Methods

### Animals and Treatment

Forty male C57BL/6J mice aged 9 weeks were purchased from the Experimental Animal Center of Xuzhou Medical University [Xuzhou, China, SCXK (Su) 2015-0009], and housed and maintained in a 12 h light/dark photoperiod with unrestricted access to water and food. After habituation to the laboratory environment for 1 week, the mice were randomly divided into four groups (*n* = 6 per group) based on their diets: (1) Control (con group) were fed a control diet (cellulose 52 g/kg, 5.2% insoluble fiber by weight) (2) M-BG group were fed a control diet supplemented with β-glucans extracted from shiitake mushroom (500 mg/kg, Yuanye Biotechnology Co., Ltd, Shanghai, China; cellulose 52 g/kg, 5.2% insoluble fiber by weight); (3) C-BG group were fed a control diet supplemented with β-glucan of curdlan (500 mg/kg, Sigma-Aldrich, St. Louis, MO, United States; cellulose 52 g/kg, 5.2% insoluble fiber by weight). The consumption of M-BG and C-BG 500 mg/kg in diet equals to a dose of ~60 mg/kg body weight for mice, as our previous mice studies showed that this dose of M-BG or C-BG rescues the gut microbiome dysbiosis (24, 25). Another study also showed that 30 mg/kg β-glucan derived from yeast effectively reduced blood glucose in rats (26). According to the dose translation formula “mice dose (mg/kg) = rat dose (mg/kg)^*^6/3” (27), therefore, the mouse dose should be 60 m/kg. (4) O-BG group mice were fed a control diet supplemented with β-glucan from oat bran (7% by food weight, β-glucan derived from OatWell^TM^ oat bran, CreaNutrition, Switzerland. 7% insoluble fiber by weight from oat bran). Based on the daily diet consumption, the dosage of β-glucans from oat bran is about 37.5 mg per mouse/day, ~1.5 g /kg body weight. The previous study had shown that β-(1,3)/(1,4)-glucans at this dose effectively modulate the gut-brain axis in mice (28, 29). Body weight and food intake were measured on the last day of each week. After 15 weeks on a diet, the cognitive behavior tests were performed, including the temporal order memory test and the Y-maze test. 4 days after behavioral testing, mice were sacrificed with CO_2_. The brain and intestine were collected and stored in −80°C for further analysis. All animal care and experiments were carried out under protocols approved by the ethics committee of Xuzhou Medical University.

### Gut Microbiota Analysis

Genomic DNA amplification, operational taxonomic units (OTUs) and 16S ribosomal RNA (rRNA) gene sequencing was performed as per our previous study (25). After the mice were euthanized, the fresh cercal contents of mice were collected into individual sterile eppendorf tubes, quickly frozen in liquid nitrogen, and then transferred into an −80°C cryogenic freezer until DNA extraction of the microbiota. Briefly, the genomic DNA from the cercal contents of mice was extracted using the E.Z.N.A. stool DNA Kit (Omega Bio-tek, Norcross, GA, U.S.) according to the manufacturer's protocols. The 16S rDNA V3-V4 region of the Eukaryotic ribosomal RNA gene was amplified by PCR (95 °C for 2 min, followed by 27 cycles at 98°C for 10 s, 62°C for 30 s, and 68°C for 30 s and a final extension at 68 °C for 10 min) using primers 341F: CCTACGGGNGGCWGCAG; 806R: GGACTACHVGGGTATCTAAT, where the barcode is an eight-base sequence unique to each sample. PCR reactions were performed in triplicate 50 μL mixture containing 5 μL of 10 × KOD Buffer, 5 μL of 2.5 mM dNTPs, 1.5 μL of each primer (5 μM), 1 μL of KOD Polymerase, and 100 ng of template DNA. Amplicons were extracted from 2% agarose gels and purified using the AxyPrep DNA Gel Extraction Kit (Axygen Biosciences, Union City, CA, U.S.) according to the manufacturer's instructions and quantified using QuantiFluor -ST (Promega, U.S.). Purified amplicons were pooled in equimolar and paired-end sequenced (2 × 250) on an Illumina platform (Gene Denovo Biotechnology Co., Ltd., Guangzhou, China). Sequence data processing were described in previous study (24, 30). Bioinformatic analysis was performed. The clean tags were clustered into operational taxonomic units (OTUs) of ≥97% similarity using UPARSE (version 9.2.64) pipeline (31). The α-diversity indices evaluating gut microbial community richness (Chao1 index) and community diversity (the Shannon index) were calculated using Mothur (32). Principal coordinate analysis (PCoA) based on OTUs unweighted unifrac distances and per mutational multivariate analysis of variance (PERMANOVA) was performed to compare the global microbiota composition after the intervention in each group at phylum and family levels, respectively. All these analyses were performed using the OmicShare tools, a free online platform for data analysis (http://www.omicshare.com/tools).

### Histological Staining and Immunohistochemistry

All animals were euthanized using CO2 inhalation. The gastrointestinal tracts were quickly removed. The colons were gently separated by cutting at the cecum-colon junction and the rectum, and immediately preserved in Carnoy's fixative (fresh anhydrous methanol: chloroform: glacial acetic acid in the ratio 60:30:10). The descending colons were fixed in Carnoy's solution (twice for 3 h). The colons were then washed in anhydrous methanol for 2 h, placed in cassettes and stored in anhydrous methanol at 4°C for further use. For the detection of colonic mucus layer thickness, the colon samples were embedded in paraffin, cut into thin sections (5 μm) and stained with Alcian blue (ab150662, Abcam, United Kingdom). In detail, the fixed colons were deparaffinized and rinsed in distilled water for 10 min, and then immersed in acetic acid for 3 min. The slides were stained in Alcian blue (pH 2.5) for 30 min at room temperature. Next, the slides were rinsed in running tap water for 2 min, and then washed using distilled water twice. Then slides were stained in Nuclear Fast Red Solution for 5 min and rinsed in running tap water for 2 min, and washed in distilled water twice. Finally, the slides were dehydrated through graded alcohols (70% alcohol, 90–95% and then 100% alcohol), cleared in xylene for 3 min for 3 times, and covered with coverslips. The thickness of the colonic mucus layer was measured using Image J.

The immunohistochemical staining has been described in our previous study (33, 34). Briefly, fixed colon tissues were embedded in paraffin and sectioned at 5 μm. The sections were deparaffinized in xylene and then in graded ethanol solutions. The sections were then washed in 3% H_2_O_2_ in methanol for 30 min. Fixed brain tissues were sectioned at 20 μm, washed 3 times for 10 min with phosphate buffer saline (PBS), and then washed in 1% H_2_O_2_ in PBS for 30 min. All sections were blocked with 5% normal goat serum and incubated with indicated primary antibodies at 4°C overnight. Primary antibodies were anti-F4/80 (ab16911, Abcam, United Kingdom, 1: 1,000 dilution) and CD206 (ab64693, Abcam, United Kingdom, 1: 1,000 dilution) for the colon and anti-Iba1 (019-19741, Wako Pure Chemical Industries, Japan, 1: 1,000 dilution) for the brain. After the primary antibody was incubated, sections were washed with PBS and then incubated with goat anti-rabbit IgG H&L (ab6702, Abcam, United Kingdom, 1: 500 dilution) for 2 h at room temperature. Finally, the sections were washed using the DAB peroxidase substrate kit (Cell Signaling Technology, Boston, MA, United States), and further counterstained with hematoxylin (Sigma-Aldrich, St. Louis, MO, United States). The sections were observed by a microscope (OLYMPUS IX51, Tokyo, Japan), and digital photographs were captured. Image J software was used to quantify the positive cells of CD206, F4/80 or Iba1in each field.

### Behavioral Tests

The temporal order memory and Y-maze tests were performed to examine dietary effects on spatial and recognition memory based on the methods previously described (25, 34). In the temporal order memory test, the experiment comprised two sample trials and one test trial with an inter-trial interval of 60 min between each trial. Mice were placed in the behavioral testing room 1 h before the test so they could acclimatize to the conditions. In each sample trial, the mice were allowed to explore two copies of the same object for 4 min; the objects were different between the two sample trials (sample trial 1: object A and A'; sample trial 2: object B and B'). During the test trial, one object from sample trial 1 (A; old familiar) and another object from sample trial 2 (B; recent familiar) were presented parallel and mice were allowed to explore the open field undisturbed for 3 min. A discrimination ratio was calculated by using the formula [(old familiar time - recent familiar time)/total exploration time]. Intact object recognition memory for temporal order was considered if the mice spent more time exploring the old familiar object compared with the recent familiar object.

For the Y-maze test, after acclimatization of the mice, the arms of the maze were labeled with different pictures. The mouse was put in the center and allowed to explore the maze undisturbed for 8 min. The number of all arm entries and alternations were recorded. The alternation was defined as the successful successive entry into each of the three arms. The alternation triplet (%) was calculated as [number of successful alternations/(total number of arms entries -2)].

### Transmission Electron Microscopy (TEM)

After being sacrificed, the mice were transcardially perfused with 4% paraformaldehyde. Brain tissues were collected and the PFC region was dissected into 1 mm^3^ of tissue blocks. Samples were fixed immediately with 2.5% glutaraldehyde at 4°C overnight. After being washed 3 times in phosphate-buffered saline (PBS), these slices were fixed in 1% osmium tetroxide, stained with 2% aqueous solution of uranyl acetate, and then dehydrated with different concentrations of ethanol and acetone gradient. Finally, the PFC blocks were embedded in epoxy resin. Ultra-thin sections (70 nm) were cut with ultra-microtome, collected on copper grids, and then stained with 4% uranyl acetate and lead citrate. Ultrastructure of synapses in the PFC region was measured under a transmission electron microscope (FEI, Portland, OR, United States). Post-synaptic density and the curvature of the synaptic interface were measured using Image J software as described previously (35, 36). The widths of the synaptic clefts (SCs) were estimated using Image J software by measuring the widest and narrowest portions of the synapse and then averaging these values (36).

### Western Blotting

Western blot assay was performed as described previously (25). Briefly, proteins were extracted from tissues of the PFC and hippocampus in cell lysis buffer containing RIPA buffer (Sigma-Aldrich, St. Louis, USA), protease inhibitor cocktail (Sigma-Aldrich, St. Louis, USA) and 1 mM PMSF (Sigma-Aldrich, St. Louis, USA), and then the concentrations quantified by BCA assay (Beyotime Biotech, Beijing, China). The same amount of proteins from each sample (20–40 μg) was separated using 10% SDS-PAGE and transferred to polyvinylidene difluoride (PVDF) membranes with a Bio-Rad electrophoresis system (Hercules, CA, USA). The membranes were blocked with 5% non-fat milk at room temperature for 2 h and then incubated overnight at 4°C with different primary antibodies. These primary antibodies included BDNF (ANT-010, Alomone Labs, Israel), PSD95 (#3450, Cell Signaling Technology, Boston, USA), β-actin (AC026, ABclonal Biotechnology Co., Ltd, USA). The membranes were then washed with PBST (0.1% Tween20) three times for 10 min and incubated with the anti-rabbit IgG conjugated with horseradish peroxidase (sc-2030, Santa Cruz Biotechnology, USA, 1:2,000 dilution) for 1 h at room temperature. Immunodetection was performed using Clarity™ ECL western blot substrate (Bio-Rad, United States) and visualized with the ChemiDoc Touch imaging system (Bio-Rad, USA).

### RNA Extraction and Quantitative Real-Time PCR

Total RNA was extracted with TRIzol (Thermo Fisher Scientific, United States) from the colon, hippocampus and the PFC. Then, 1 μg RNA was reverse-transcribed to cDNA using a high-capacity cDNA reverse transcription kit (Takara, Japan). The cDNA was used for quantitative Real-Time PCR using the SYBR GREEN Master Mix (TaKaRa, Japan) on a real-time PCR detection system (Bio-Rad, United States). The relative mRNA levels were calculated using the comparative CT method (2^−Δ*ΔCt*^) and normalized by β-actin mRNA levels. All primers are listed in Supplementary Table 1.

### Statistical Analysis

The data are presented as mean ± SEM. Statistical analysis was performed using the one-way analysis of variance (ANOVA) followed by the *post-hoc* Tukey test for comparisons among the groups by SPSS (version 20, IBM Corporation, Chicago, IL, United States). The ANOVA analysis provided *F* (df) and *P*-values. A *P*-value < 0.05 was considered to be statistically significant, marked with ^*^ (^*^P < 0.05, ^**^P < 0.01, ^***^P < 0.001). Pearson's correlation analysis was used to examine the relationships between two parameters.

## Results

### β-Glucans Enhanced Temporal Order Recognition Memory and Synaptic Ultrastructure

**Figure 1:**
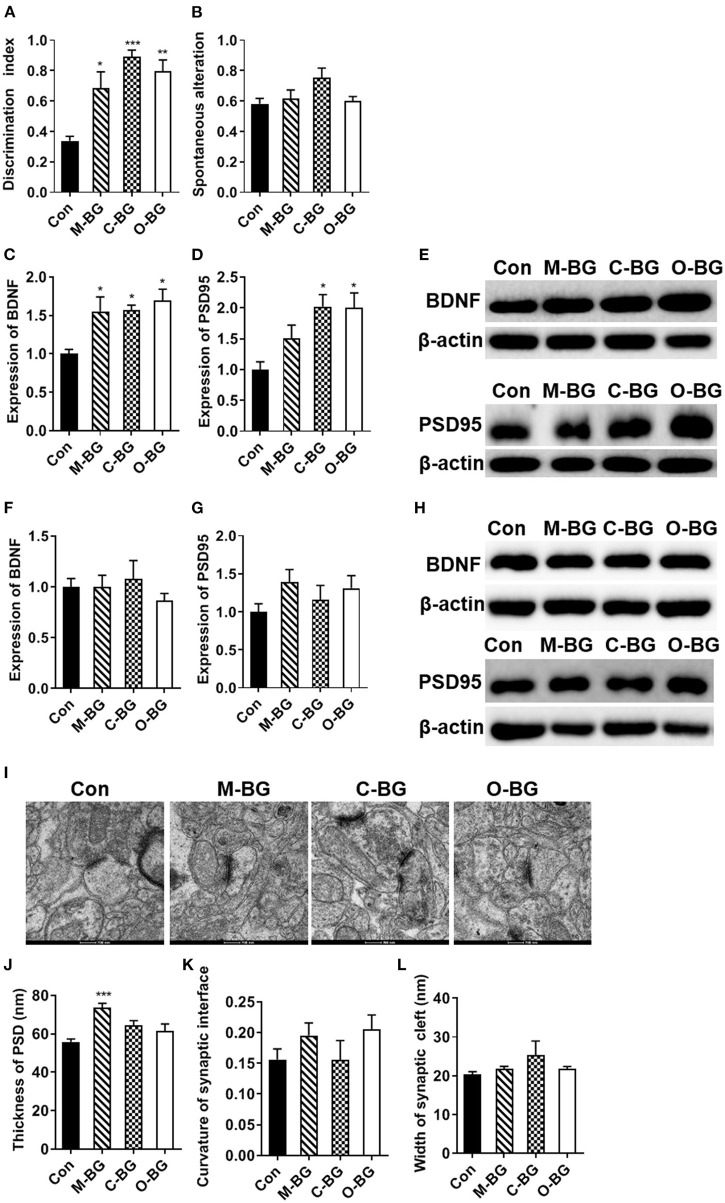
Chronic β-glucans supplementation improved cognition and synaptic plasticity. **(A)** Discrimination index in the temporal order memory test, *n* = 6. **(B)** Proportion of correct alternations in the Y-maze test, *n* = 6. **(C–E)** The protein expression levels of BDNF and PSD95 in the PFC, *n* = 4. **(F–H)** The protein expression levels of BDNF and PSD95 in the hippocampus, *n* = 4. **(I)** Electron micrograph of synaptic ultrastructure in the PFC, *n* = 6. **(J–L)** Image analysis of the thickness of PSD **(J)**, the curvature of the synaptic interface **(K)**, and width of synaptic cleft **(L)** in the PFC. Data were presented as mean ± SEM. *P < 0.05 vs. Control, **P < 0.01 vs. Control, ***P < 0.001 vs. Control. Scale bar: 200 nm.

In the temporal order memory test, 15 weeks of three β-glucan supplementations significantly elevated the discrimination index that measures recognition memory, compared to the control diet [*F*_(3,20)_ = 11.917, *P* < 0.001, Figure 1A]. In the Y-maze test, there was no difference in the proportion of spontaneous alteration among the four groups [*F*_(3,20)_ = 2.719, *P* = 0.72, Figure 1B], suggesting that short-term spatial working memory was not affected by β-glucan supplementations. Synaptic plasticity is considered to be one of the main cellular mechanisms for cognition, including memory performance (37). To explore the potential mechanisms of dietary β-glucans improving the cognition capability, the proteins modulating synaptic plasticity and synaptic ultrastructure were examined. Our results showed that three β-glucan supplements increased the level of brain-derived neurotrophic factor (BDNF) [*F*_(3,12)_ = 5.801, *P* = 0.011, Figures 1C,E]. The post-synaptic protein 95 (PSD95) level was also increased in the PFC of C-BG and O-BG groups [*F*_(3,12)_ = 5.953, *P* = 0.010, Figures 1D,E], but this did not reach significance in the M-BG group. While in the hippocampus, the three types of dietary β-glucans did not affect the BDNF [*F*_(3,12)_ = 0.521, *P* = 0.676] and PSD95 levels [*F*_(3,12)_ = 1.176, *P* = 0.360] (Figures 1F–H). TEM results showed that the thickness of PSD was significantly increased in the M-BG group compared with the control group [*F*_(3,20)_ = 8.254, *P* = 0.001, Figures 1I,J]. β-glucan supplementations had no effect on the curvature of synaptic interface [*F*_(3,19)_ = 1.184, *P* = 0.342, Figure 1K] and width of synaptic clefts [*F*_(3,20)_ = 1.282, *P* = 0.308, Figure 1L]. In addition, no significant difference in body weight was detected among the four groups (Supplementary Figure 1) and there was no difference in cumulative food intake among the four groups (Supplementary Figure 2).

### The Effects of β-Glucans on Microglia Number, Complement C3, and Cytokines Expression

**Figure 2:**
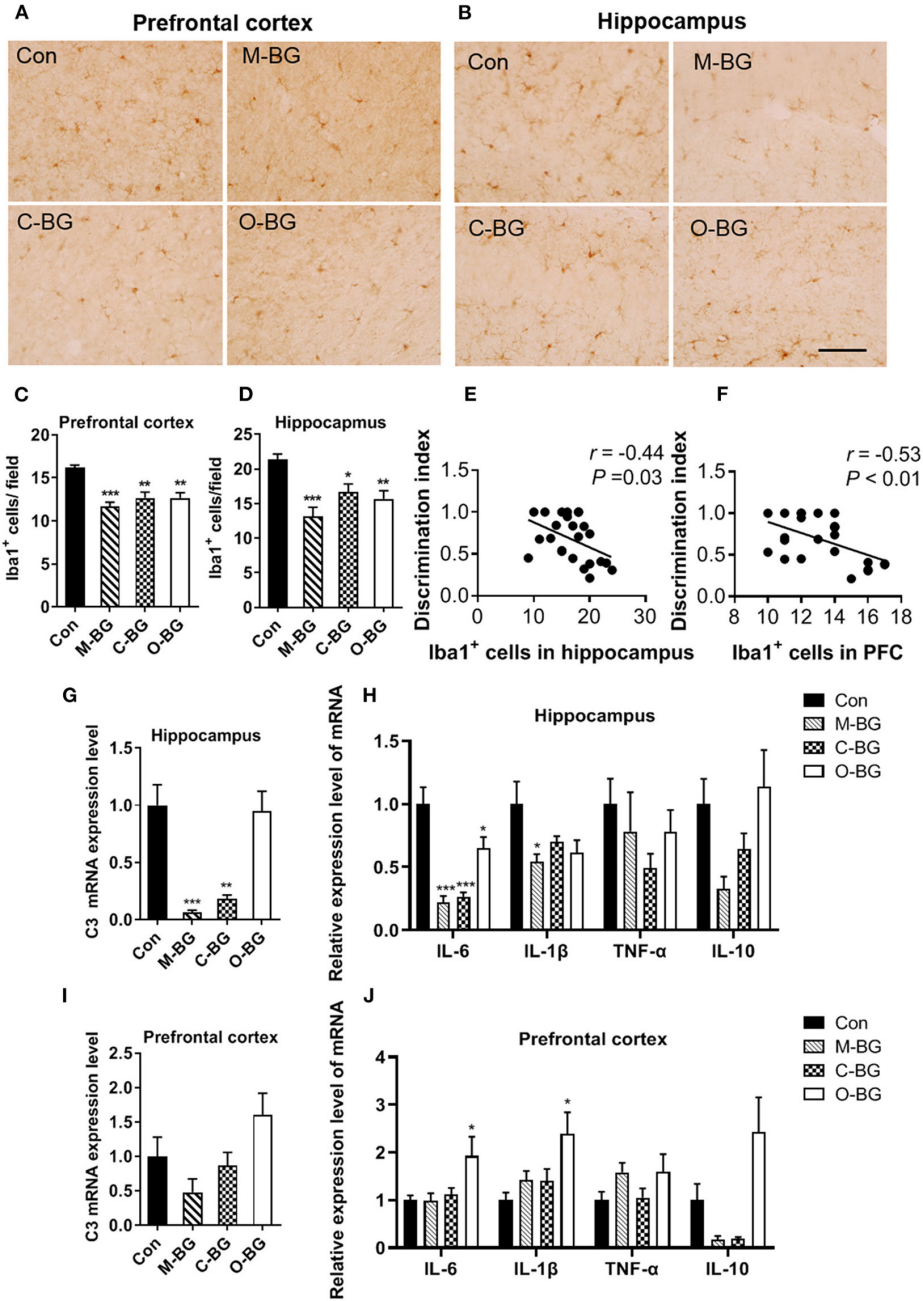
Effects of dietary β-glucans on microgliosis and neuroinflammation of mice. **(A)** Immunohistochemical staining of Iba1 positive cells in the PFC of mice, *n* = 6. **(B)** Immunohistochemical staining of Iba1 positive cells in the hippocampus of mice, *n* = 6. **(C)** Quantification of Iba1 positive cells in the PFC of mice. **(D)** Quantification of Iba1 positive cells in the hippocampus of mice. **(E)** Pearson's correlation analysis between Iba1 positive cells in the hippocampus and discrimination index. **(F)** Pearson's correlation analysis between Iba1 positive cells in the PFC and discrimination index. **(G)** mRNA expression of C3 in the hippocampus of mice, *n* = 6. **(H)** mRNA expression of IL-6, IL-1β, TNF-α, and IL-10 in the hippocampus of mice, *n* = 6. **(I)** mRNA expression of C3 in the PFC of mice, *n* = 5–6. **(J)** mRNA expression of IL-6, IL-1β, TNF-α, and IL-10 in the PFC of mice, *n* = 6. Data were presented as mean ± SEM. *P < 0.05 vs. Control, **P < 0.01 vs. Control, ***P < 0.001 vs. Control. Scale bar: 40 μm.

Microglia, as an innate immune cell of the central nervous system, plays an important role in regulating synaptic pruning and remodeling and cognitive behaviors (38). Next, we examined the effects of three types of dietary β-glucans on microglia number, complement C3 and cytokines expression in the PFC and hippocampus. We found that numbers of Iba1 (the marker of activated microglia) positive cells were significantly reduced in all three types of dietary β-glucan supplement groups compared to that of the control group in the PFC [*F*_(3,20)_ = 13.421, *P* < 0.001, Figures 2A,C] and the hippocampus [*F*_(3,20)_ = 9.412, *P* < 0.001, Figures 2B,D]. The Pearson's correlation analysis showed that the number of Iba1 positive cells in the hippocampus and the PFC were both negatively correlated with the discrimination index in the temporal order memory test (*r* = −0.44, *P* = 0.03, Figure 2E; *r* = −0.53, *P* < 0.01, Figure 2F). Three types of dietary β-glucans differentially influenced C3 expression in the PFC and hippocampus. The β-glucans from mushroom and curdlan supplement greatly decreased the C3 expression in the hippocampus [*F*_(3,20)_ = 15.047, *P* < 0.001, Figure 2G], but not in the PFC (Figure 2I) compared with the control group. In the hippocampus, the mRNA expression of IL-6 was reduced in all three dietary β-glucan supplementation groups [*F*_(3,20)_ = 18.654, *P* < 0.001, Figure 2H], and IL-1β mRNA expression significantly decreased only in the M-BG group [*F*_(3,20)_ = 3.389, *P* = 0.038, Figure 2H]. TNF-α and IL-10 mRNA expression in the hippocampus was not significantly altered in the three groups (Figure 2H). In the PFC, M-BG, C-BG did not affect cytokines expression (Figure 2J), while O-BG supplementation increased IL-6 [*F*_(3,20)_ = 3.792, *P* = 0.027, Figure 2J] and IL-1β [*F*_(3,20)_=4.361, *P* = 0.016, Figure 2J] mRNA expression compared with the control group. These results indicated that all three types of β-glucans supplement inhibited the activation of microglia but had differential effects on C3 and cytokines expression in the PFC and hippocampus.

### β-glucans Promoted Macrophage M2 Polarization and Increased IL-10 in the Colon

**Figure 3:**
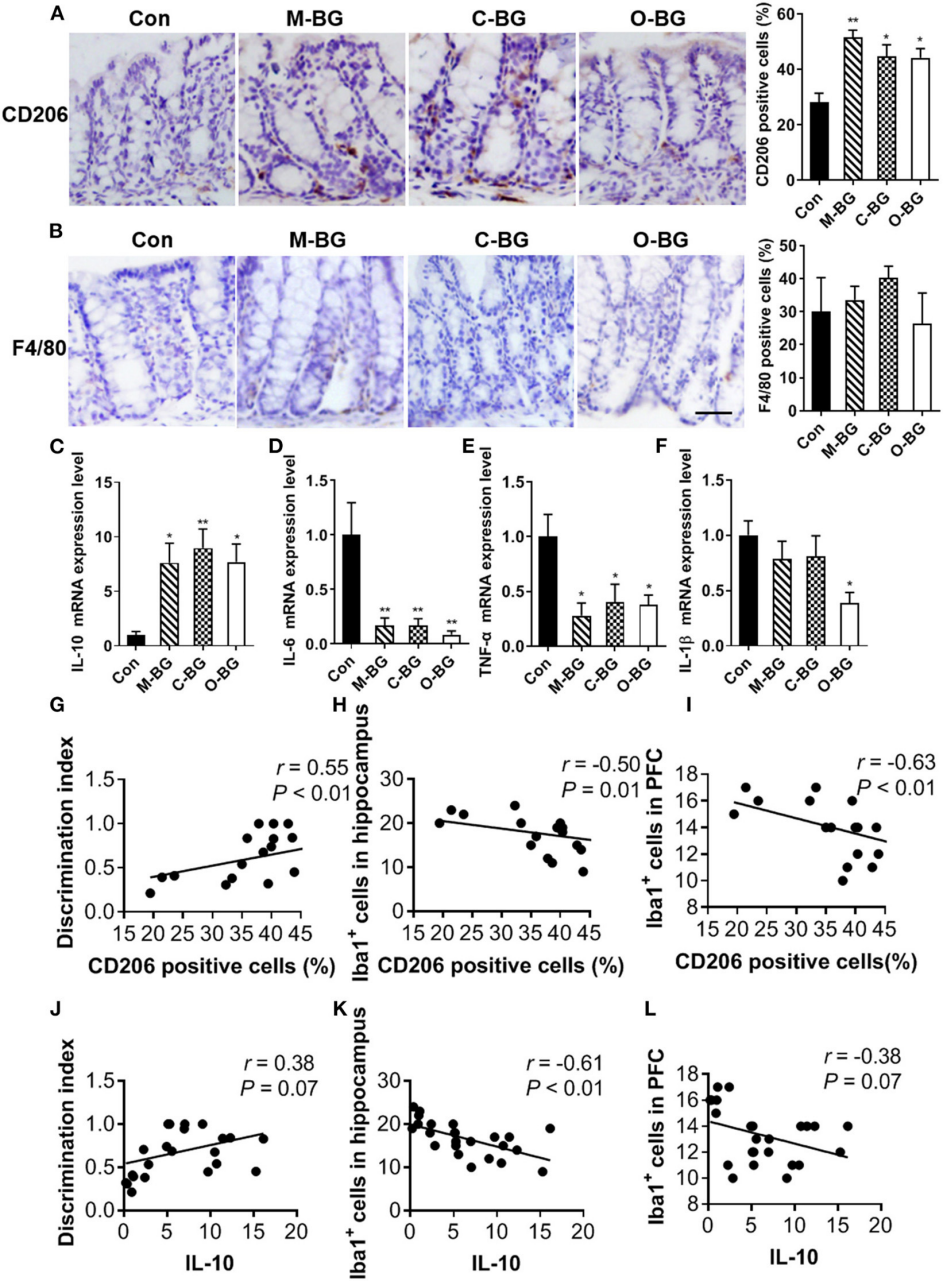
β-glucans promoted macrophage M2 polarization in the colon. **(A)** Immunohistochemical staining and quantification of colonic CD206-positive cells. **(B)** Immunohistochemical staining and quantification of colonic F4/80-positive cells. **(C–F)** mRNA expression levels of IL-10 **(C)**, IL-6 **(D)**, TNF-α **(E)**, IL-1β **(F)** in the colon of mice. **(G–I)** Pearson's correlation analysis between CD206 positive cells in colon and discrimination index **(G)**, Iba1^+^cells in the hippocampus **(H)** and in the PFC **(I)**. If *P* < 0.05 for significant correlations, R is noted. **(J–L)** Pearson's correlation analysis between IL-10 mRNA expression in colon and discrimination index **(J)**, Iba1^+^cells in the hippocampus (K) and Iba1^+^cells in the PFC **(L)**. If *P* < 0.05 for significant correlations, R is noted. All *n* = 6. Data were presented as mean ± SEM. *P < 0.05 vs. Control, **P < 0.01 vs. Control, ***P < 0.001 vs. Control. Scale bar: 80 μm.

The gut-brain axis plays an important role in cognition (39). We also examined the effects of β-glucans on the colonic macrophage polarization and cytokines expression. Our results showed that M-BG, C-BG and O-BG supplementations significantly increased the positive cell number of CD206 (M2 macrophage marker) in the colon compared to control mice [*F*_(3,20)_ = 8.463, *P* = 0.001, Figure 3A]. However, the number of F4/80 positive cells (total macrophages) was not affected by β-glucan supplementations [*F*_(3,20)_ = 0.635, *P* = 0.601, Figure 3B]. The mRNA levels of the anti-inflammatory cytokine IL-10 (produced by M2 macrophages) were dramatically increased in M-BG, C-BG and O-BG groups compared with the control group [*F*_(3,20)_ = 5.716, *P* = 0.005, Figure 3C]. Furthermore, pro-inflammatory cytokines IL-6 and TNF-α were both decreased in three β-glucans groups [*F*_(3,20)_ = 7.612, *P* = 0.001, Figure 3D; *F*_(3,20)_ = 4.834, *P* = 0.011, Figure 3E]. The IL-1β mRNA was significantly decreased by β-glucans from oat bran, but not mushroom and curdlan in the colon (*P* = 0.038, Figure 3F). Consistent with the result of previous research demonstrates there is an association between gut and brain (39), here, the Pearson's correlation analysis showed that the CD206 positive cells and IL-10 expression in the colon were positively correlated with the discrimination index (*r* = 0.55, *P* < 0.01, Figure 3G; *r* = 0.38, *P* = 0.07, Figure 3J), and negatively correlated with Iba1^+^ cells in the hippocampus (*r* = −0.50, *P* = 0.01, Figure 3H; *r* = −0.61, *P* < 0.01, Figure 3K) and PFC (*r* = −0.63, *P* < 0.01, Figure 3I; *r* = −0.38, *P* = 0.07, Figure 3L). Collectively, data described above indicated that three dietary β-glucan supplementations promoted M2 macrophage polarization in the colon, which may be associated with cognitive behavior and microglia status in the hippocampus and PFC.

### β-glucans Had Different Effects on the Thickness of Colon Mucus and Gut Microbiota

**Figure 4:**
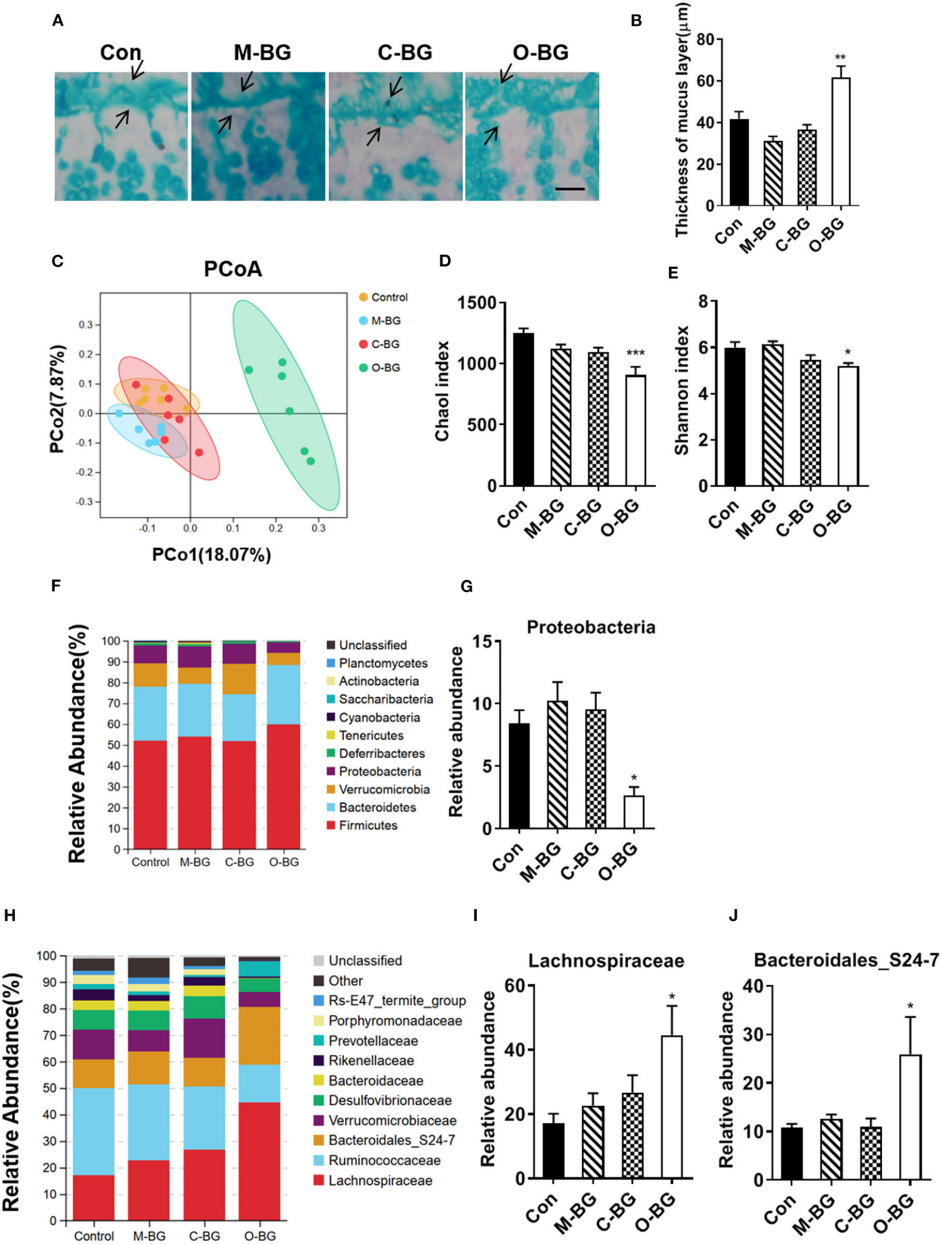
β-glucans had different effects on gut microbiota. **(A,B)** Alcian blue staining for colon and quantification of colonic mucus layer thickness, *n* = 6. **(C)** Principal coordinates analysis plot of unweighted UniFrac distances, *n* = 6. **(D)** Richness (Chao1 index) of gut microbial communities, *n* = 6. **(E)** Diversity (Shannon index) of gut microbial communities, *n* = 6. **(F)** The relative abundance of bacteria at the phylum level, *n* = 6. **(G)** Relative abundance of Proteobacteria, *n* = 5–6. **(H)** The relative abundance of bacteria at the family level, *n* = 6. **(I,J)** Relative abundance of Lachnospiraceae and Bacteroidales_S24-7 families, *n* = 5–6. Data were presented as mean ± SEM. *P < 0.05 vs. Control, **P < 0.01 vs. Control, ***P < 0.001 vs. Control. Scale bar: 80 μm.

The intestinal mucus and gut microbiota have been reported to be involved in the gut-brain axis (40, 41). Next, we examined the effects of three types of β-glucans on the colon mucus and gut microbiota profiles. The thickness of colon mucus was significantly increased in the colon of mice with supplementations of β-glucans from oat bran, but not from the mushroom and curdlan [*F*_(3,20)_ = 13.072, *P* < 0.001, Figures 4A,B]. Furthermore, we found that β-glucans from oat bran was the most prominent one to alter the gut microbiota profile with Illumina MiSeq sequencing of 16S rDNA genes. Principal coordinate analysis (PCoA) based on OTUs unweighted unifrac distances confirmed the heterogeneity of gut microbiota among the four treatment groups, in which a clear separate cluster was observed between the O-BG group and the other two groups as well as the control group (Figure 4C). For α diversity, O-BG group had lower species richness (Chao1 index) [*F*_(3,20)_ = 9.408, *P* < 0.001, Figure 4D], and lower species diversity (Shannon index) [*F*_(3,20)_ = 6.105, *P* = 0.004, Figure 4E] than the control group. The key phylotypes were distributed among four bacteria phyla, including Firmicutes, Bacteroidetes, Verrucomicrobia and Proteobacteria (Figure 4F). Furthermore, the proportion of Proteobacteria at the phylum level was remarkably decreased in mice with the supplementation of β-glucan from oat bran compared with the control group [*F*_(3,19)_ = 7.270, *P* = 0.002, Figure 4G]. At the family level, β-glucan from oat bran increased the abundance of Lachnospiraceae and Bacteroidales_S24-7 families (Figure 4H) [*F*_(3,20)_ = 4.175, *P* = 0.019, Figure 4I; *F*_(3,19)_ = 4.010, *P* = 0.023, Figure 4J]. Therefore, among three types of β-glucans, β-glucan from oat bran showed significant effects on the enhancement of intestinal mucus and modulation of gut microbiota.

## Discussion

It is reported that dietary fiber intake consumption is positively correlated with cognition in the middle-aged and elderly population (1, 2, 42). Mushroom, curdlan and oat bran provide a good source of dietary fiber and their main element is β-glucan. β-glucans of different origins and different chemical structure exhibit their unique beneficial effect on the intestine, immune system and central nervous system. The present study provides the first comparison of the potency of three main types of β-glucans on cognitive function, intestinal immune response and gut microbiota profiles. Our results showed that long-term supplementation of β-glucans from mushroom, curdlan and oat bran enhanced temporal order recognition memory, synaptic proteins levels, and ultrastructure in mice. These β-glucans inhibited the microgliosis in the PFC and hippocampus, the important brain regions for cognition. Besides, all three types of β-glucan supplementations promoted macrophage M2 polarization, increased IL-10 and decreased pro-inflammation cytokines in the colon. Particularly, β-glucan from oat bran augmented the integrity of the colonic mucus barrier and shifted the gut microbiota compositions.

Deficits in the temporal order memory of items or events in a sequence are currently considered to be age-related dysfunction in older humans (43, 44) and a cognitive-behavioral marker for neurodegenerative diseases, including Alzheimer's Disease (45) and Parkinson's Disease (46). Previous clinical studies found that dietary fiber intake is positively correlated with cognition in elderly people of Korea (*n* = 449) (1) and in institutionalized older people from Madrid (*n* = 178) (42). In a study of Australians in mid-aged and older (*n* = 617), usual consumption of higher fiber or multigrain bread is associated with better cognitive function compared to white bread (2). β-glucan is a polysaccharide in the form of fiber and the main element of fiber in oats, barley, yeast and mushrooms. In this study, we found that chronic supplementation of three types of β-glucans from mushroom, curdlan and oat bran increased temporal order memory in mice. These findings suggest that β-glucans as the main bioactive compounds in the fiber or fiber-rich food, may contribute to cognition enhancement and delay neurodegeneration. Furthermore, previous research, including ours, reported that a variety of β-glucan supplementations improved cognitive decline in rodents induced by obesity (25, 34, 36), scopolamine (47) or Aβ1–42 (48). The effects of β-glucans in improving cognition are evident in disease models; however, it is not clear whether β-glucans directly enhance the cognitive function or indirectly through treating other pathology that causes a cognitive deficit in these disease models. In the present study, we demonstrated β-glucans from oats bran, mushroom, curdlan, representing three classical β-glucans, β-(1,3)/(1,4)-glucan, β-(1,3)/(1,6)-glucan and β-(1,3)-glucan, have the ability to increase the temporal order memory in normal mice, suggesting that β-glucans are direct cognition enhancers.

The potential molecular mechanisms about how β-glucans enhance cognition may be attributed to increasing synaptic proteins and strengthening synaptic ultrastructure. In the present study, supplementation of three types of β-glucans from mushroom, curdlan and oat bran increased BDNF level in the PFC, which is important for cognitive function, especially for temporal order memory (49). It is known that BDNF signaling facilitates synaptic plasticity and neural structure and thus plays key roles in learning and memory processes (50). Dysfunctional BDNF signaling in the PFC, but not in the hippocampus, results in the PFC-dependent cognitive deficits in the subchronic PCP-induced rodent model of schizophrenia (51). Previous studies also show that BDNF expression is reduced in the cortex of Alzheimer's disease patients (52). Therefore, β-glucans increasing BDNF in the PFC may contribute to the enhancement of cognition and preventing neurodegeneration and reducing the risk of Alzheimer's disease. PSD95 is a major scaffolding protein located within the post-synaptic region of excitatory synapses (53). PSD95 deficiency disrupts PFC synaptic function and related cognitive behavior in mice (54). Our findings that supplementation of β-glucans increased PSD95 protein level and PSD thickness of synaptic ultrastructure in the PFC might be the structural alterations that are associated with cognitive enhancement.

Microglia activation mediates neuro inflammatory reaction, causing progressive neurodegeneration and cognitive impairment (55, 56). We found that three β-glucan supplementations significantly attenuated the number of Iba-1 positive cells in the hippocampus and PFC. Iba-1 is essential for the morphological changes from quiescent ramified microglia to activated amoeboid microglia (57, 58). Furthermore, we found that the supplementation of β-glucans from mushroom and curdlan, significantly inhibited the C3 level in the hippocampus. It is reported that complement-mediated neuro inflammation is associated with degenerative changes after traumatic brain injury, while inhibition of C3 activation reduces neuroinflammation (59). However, the underneath molecular mechanism of β-glucans in inhibition of C3 and microglia accumulation requires further investigation. Besides, microglia are the major source of inflammatory cytokines in CNS to induce neuro inflammation, the main mechanism underlying various neurodegenerative diseases (55, 56). It is reported that pro-inflammatory cytokines (IL-6 and IL-1β) were higher in patients with Alzheimer's type dementia, compared to healthy controls (60). We found that all three β-glucans suppressed IL-6 mRNA expression in the hippocampus. IL-6 is considered to be an index of chronic inflammation since IL-6 had a longer half-life than TNF-α and IL-1β (61). Therefore, our data suggest that β-glucans might be capable of protecting chronic neuro inflammation in the hippocampus. Interestingly, we did not find that three β-glucans inhibit TNF-α, IL-6 and IL-1β mRNA expression in the PFC. The highest levels of both IL-6 and IL-6 receptor transcripts were detected in the adult rat hippocampus (62). The previous study had shown that IL-6 blockade sufficiently restores hippocampal neurogenesis when neural progenitors are exposed to a conditioned medium of activated microglia (63). Transgenic mice with excessive IL-6 displayed impaired avoidance learning, whereas the administration of IL-6 receptor antagonists prevents the decreases in hippocampal long-term potentiation and neurogenesis (64). Overall, these findings suggest that β-glucans-induced inhibition of activated amoeboid microglia, complement and proinflammatory cytokines may contribute to the enhancement of cognitive ability.

Emerging evidence indicates a connection between the gut and the brain. For example, patients with inflammatory bowel disease (IBD) exhibit harmful neuropsychological repercussions, although the exact pathophysiological mechanisms are not fully clarified (65). Recently, a large-sample size longitudinal study shows that the incidence of dementia was significantly elevated among patients with IBD (66). In the present study, the number of M2 macrophages was increased in the colon of mice supplemented with three types of β-glucans individually. In a steady-state or healthy colon, most resident macrophages are M2 type that expresses CD206 and produce IL-10 (67). We found that three types of β-glucans all increased the anti-inflammatory cytokine IL-10 level and inhibited the pro-inflammatory cytokines TNF-α and IL-6 in the colon. Therefore, these findings suggest that β-glucan supplements are beneficial for the maintenance of the health status of the colon. It is reported that circulating IL-10 level is associated with higher measures of cognitive function (68). Peripheral administration of IL-10 rescues depression-associated learning and memory deficits in mice (69). IL-10(-/-) mice exhibit a more prominent reduction in cognitive function after LPS challenging compared to IL-10(+/+) mice. These IL-10(-/-) mice also produce more pro-inflammatory cytokines IL-1β, IL-6 and TNF-α in the plasma, suggesting IL-10 inhibits cognitive decline via its propensity to mitigate inflammation (70). In line with these studies, our results revealed that colon IL-10 levels and the percentage of CD206 positive cells were positively correlated with the temporal order memory and negatively correlated with Iba1 positive cells numbers in the PFC and hippocampus. These findings provide evidence that β-glucan supplements promote M2 macrophage shift and increase anti-inflammatory IL-10 expression, and consequently consolidate the healthy status of the colon and improve cognition.

We also found that β-glucans from oat bran, but not from the mushroom and curdlan, greatly shifted the gut microbiota and increased the thickness of colon mucus. Oat β-glucan is mixed-linkage β-(1,3)/(1,4)-glucans (MLG) (71). Previous studies demonstrated that the MLG is the specific site of hydrolysis by the MLG utilization locus, BoGH16_MLG_, of Bacteroidetes lower taxa, such as *Bacteroides ovatus* (72). In the present study, we found that the supplementation of β-glucan from oat bran significantly increased Bacteroidales_S24-7 family. We speculated that this alteration in gut microbiota might contribute the cognitive improvement as a recent study reports that OTUs belonging to Bacteroidales_S24-7 are positively correlated with cognitive and social behaviors in mice (73). Moreover, the abundance of Bacteroidales_S24-7 is significantly decreased in a mouse model of Alzheimer's disease (74). In addition, we also found that oat β-glucan supplement also increases the level of the Lachnospiraceae families, which might be devoted to the enhancement of the gut integrity because probiotic *Lactobacillus paracasei* administration restored the gut integrity in the diabetic rat model (75). Furthermore, we show that oat β-glucan supplementation significantly decreased the abundance of phylum Proteobacteria. The amount of Microbiota belonging to the phylum Proteobacteria has been positively associated with Alzheimer's disease, neurodegeneration and the related neuroinflammatory status. For example, gut microbiota shifts toward higher abundances of Proteobacteria in the transgenic APP/PS1 mouse model of Alzheimer's disease (76). The aging population has increased the phylum Proteobacteria and the abundance of several bacteria belonging to the Proteobacteria is positively correlated with IL-6 and IL-8 (77). Excessive growth of Proteobacteria causes colitis, resulting in the occurrence of neuroinflammation and cognitive decline in mice induced by Escherichia coli K1 (78). While probiotics, *Lactobacillus gasseri NK109*, decrease the Proteobacteria and reverse all these dysfunctions (78). Taken together, these findings suggest that suppressed Proteobacteria population by prebiotic oat β-glucan or probiotics may improve the gut-brain axis and enhance cognition. Interestingly, we find β-glucan of mushrooms and curdlan had no effect on gut microbiota. The β-glucan of mushrooms is β-(1,3)/(1,6)-glucan and curdlan is composed of linear β-(1,3)-glucan. Therefore, our results indicate that mixed-linkage β-(1,3)/(1,4)-glucans may be the main important target of gut microbiota, and potentially resulting in compositional and functional shifts in the gut microbiota. In this study, we did not find the effect of M-BG and C-BG (60 mg/kg) on the intestinal flora in normal mice, though our previous studies had revealed that 15 weeks supplementation of M-BG or C-BG at the same dose prevented the gut microbiota dysbiosis in mice model of high fat diet-induced non-alcoholie fatty liver disease (24) and cognitive decline (25), respectively. This suggests that β-(1,3)/(1,6)-glucans or β-(1,3)-glucans from mushroom and curdlan may have effects as prebiotics to correct disturbed gut microbiota, but preserve and do not change the normal gut microbiota homeostasis. It is also plausible that the dose of M-BG or C-BG (60 mg/kg) might not be high enough to regulate normal gut microbiome. Previously, yeast β-glucan at 100 mg/kg regulates gut microbiota as prebiotics in Aβ 1-42-induced AD-like mice (48). To overcome the limitation of the low dosage of M-BG or C-BG, we will increase the dose of these β-glucan up to 100 mg/kg in the future study, as this is a safe dose that did not induce toxic effect (79).

## Conclusions

In the present study, for the first time, we demonstrated the difference and similarity of the three β-glucans on the regulation of the microbiota-gut-brain axis, which lay the basis for further research and clinical applications. To a broader view, our finding about the beneficial effect of long-term supplementation of β-glucans on cognitive function and gut microbiome could be extended to apply into a disease model of cognitive decline. Our future studies will determine which type of β-glucans exhibit the best beneficial effect to improve cognitive impairment in a senescence-accelerated mouse prone 8 (SAMP8) mouse model with rapid cognitive decline, APP transgenic mouse models for Alzheimer's disease and high-fat diet-induced obese mice with cognition decline.

## Data Availability Statement

All 16S rRNA raw data were submitted to the NCBI Sequence Read Archive (SRA) Database with the accession number: PRJNA801759.

## Ethics Statement

The animal study was reviewed and approved by the Ethics Committee of Xuzhou Medical University (Xuzhou, China, SCXK (Su) 2015-0009).

## Author Contributions

MH, PZ, RW, and YY designed the research study. MH, PZ, RW, MZ, and NP performed the research. MH, PZ, RW, and XG analyzed the data. MH, PZ, and YY wrote the manuscript. XC, XL, X-FH, and YY reviewed the manuscript. All authors approved the manuscript.

## Funding

This work was supported by the Starting Foundation for Talents of Xuzhou Medical University [Nos. D2017007 and D2018003]; the National Natural Science Foundation of China [Nos. 81700794, 81971179, and 82071184]; the Natural Science Foundation of Jiangsu Province [No. BK20191463]; the Jiangsu Graduate Innovation Program [No. KYCX20_2469].

## Conflict of Interest

The authors declare that the research was conducted in the absence of any commercial or financial relationships that could be construed as a potential conflict of interest.

## Publisher's Note

All claims expressed in this article are solely those of the authors and do not necessarily represent those of their affiliated organizations, or those of the publisher, the editors and the reviewers. Any product that may be evaluated in this article, or claim that may be made by its manufacturer, is not guaranteed or endorsed by the publisher.
