# Supplementary figures and images for: Three Different Types of β-Glucans Enhance Cognition: The Role of the Gut-Brain Axis
Source: Front Nutr. 2022 Mar 3;9:848930. doi: 10.3389/fnut.2022.848930 (PMC8927932; doi:10.3389/fnut.2022.848930)

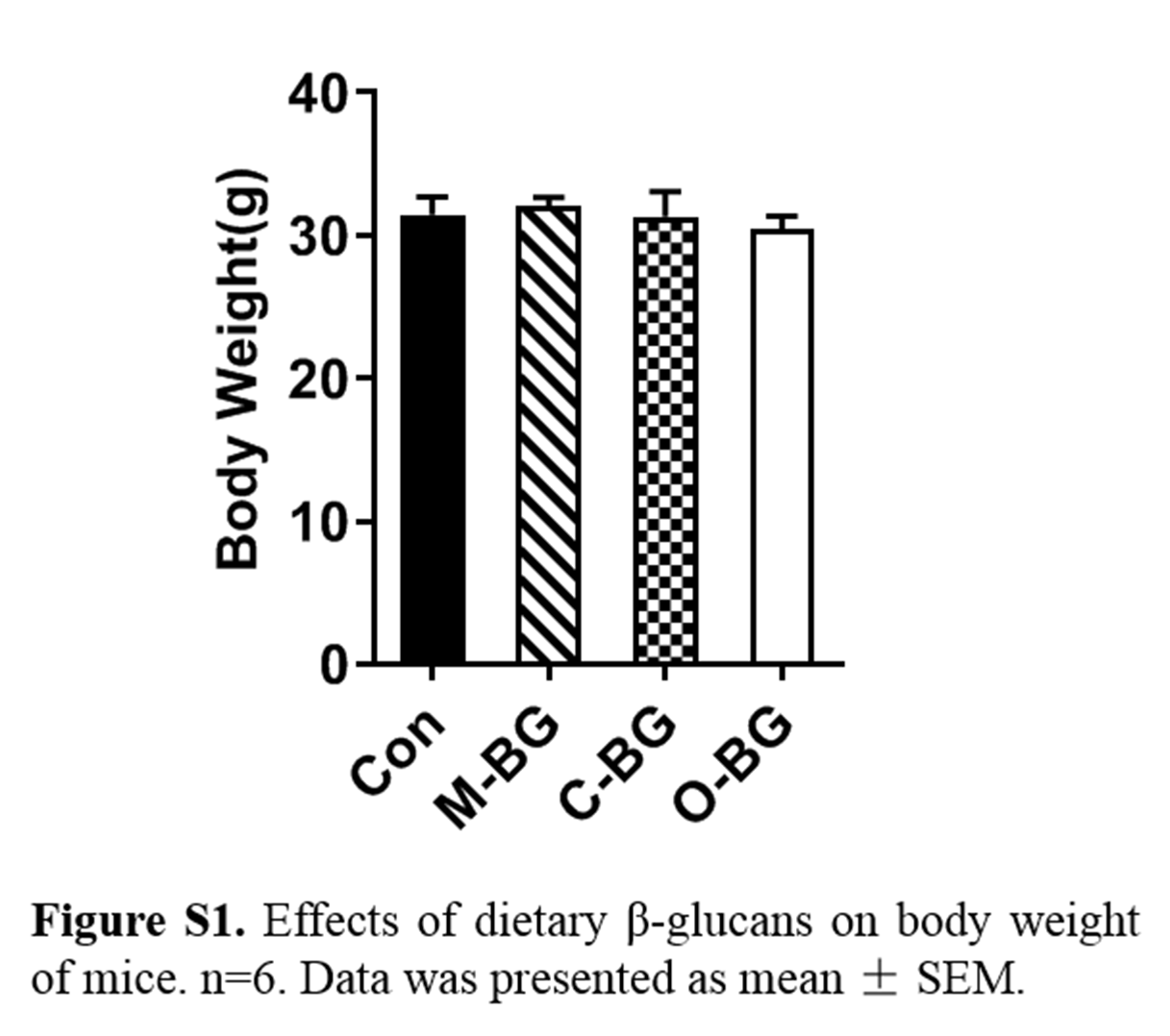

Supplement: Supplementary file 1 [file Image_1.TIF]

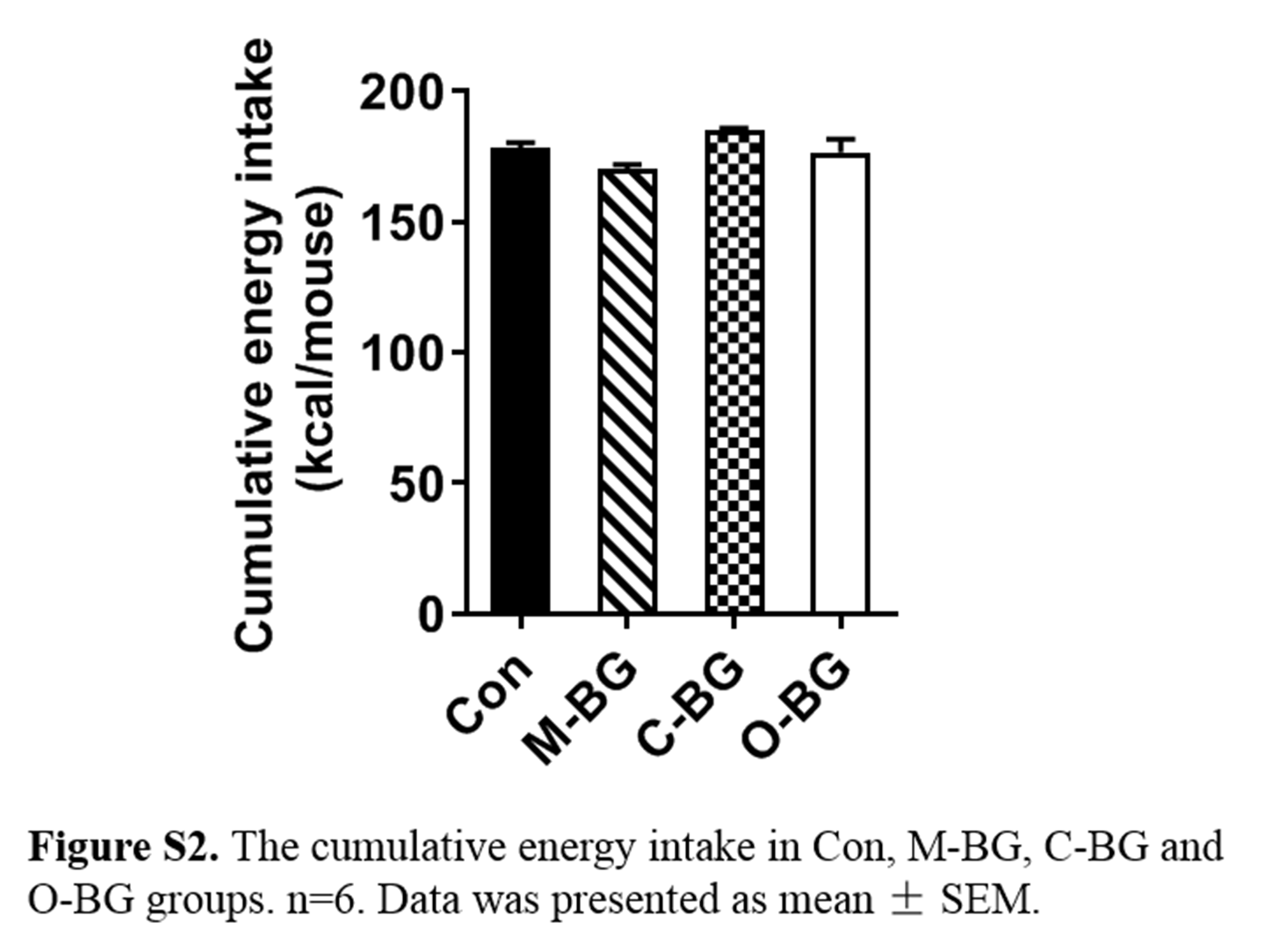

Supplement: Supplementary file 2 [file Image_2.TIF]
