# Supplementary material for: Three Different Types of β-Glucans Enhance Cognition: The Role of the Gut-Brain Axis
Source: Front Nutr. 2022 Mar 3;9:848930. doi: 10.3389/fnut.2022.848930 (PMC8927932; doi:10.3389/fnut.2022.848930)
Supplement: Supplementary file 3 [file Table_1.DOCX]

**Table S1.** Primers used for Quantitative Real-Time PCR

| **Mouse Gene** | **Forward Primer (5’**→**3’)** | **Reverse Primer (5’**→**3’)** |
| --- | --- | --- |
| TNF-α | CTTGTTGCCTCCTCTTTTGCTTA | CTTTATTTCTCTCAATGACCCGTAG |
| IL-1β | TGGGAAACAACAGTGGTCAGG | CTGCTCATTCACGAAAAGGGA |
| IL-6 | TCACAGAAGGAGTGGCTAAGGACC | ACGCACTAGGTTTGCCGAGTAGAT |
| IL-10 | GGAAGACAATAACTGCACCCACT | CAACCCAAGTAACCCTTAAAGTCC |
| β-actin | TGAGAGGGAAATCGTGCGTGAC | GCTCGTTGCCAATAGTGATGACC |
